# Orangutans show active voicing through a membranophone

**DOI:** 10.1038/s41598-019-48760-7

**Published:** 2019-08-23

**Authors:** Adriano R. Lameira, Robert W. Shumaker

**Affiliations:** 1 School of Psychology and Neuroscience, University of St. Andrews, St Andrews, UK; 2 Department of Anthropology, Durham University, Durham, UK; 3 Indianapolis Zoo, Indianapolis, USA; 4 Krasnow Institute for Advanced Studies, George Mason University, Fairfax, USA; 5 Anthropology Department, Indiana University, Bloomington, USA

**Keywords:** Psychophysics, Biological anthropology, Human behaviour, Animal behaviour

## Abstract

Active voicing – voluntary control over vocal fold oscillation – is essential for speech. Nonhuman great apes can learn new consonant- and vowel-like calls, but active voicing by our closest relatives has historically been the hardest evidence to concede to. To resolve this controversy, a diagnostic test for active voicing is reached here through the use of a membranophone: a musical instrument where a player’s voice flares a membrane’s vibration through oscillating air pressure. We gave the opportunity to use a membranophone to six orangutans (with no effective training), three of whom produced a priori novel (species-atypical) individual-specific vocalizations. After 11 and 34 min, two subjects were successful by producing their novel vocalizations into the instrument, hence, confirming active voicing. Beyond expectation, however, within <1 hour, both subjects found opposite strategies to significantly alter their voice duration and frequency to better activate the membranophone, further demonstrating plastic voice control as a result of experience with the instrument. Results highlight how individual differences in vocal proficiency between great apes may affect performance in experimental tests. Failing to adjust a test’s difficulty level to individuals’ vocal skill may lead to false negatives, which may have largely been the case in past studies now used as “textbook fact” for great ape “missing” vocal capacities. Results qualitatively differ from small changes that can be caused in innate monkey calls by intensive months-long conditional training. Our findings verify that active voicing beyond the typical range of the species’ repertoire, which in our species underpins the acquisition of new voiced speech sounds, is not uniquely human among great apes.

## Introduction

Language defines the primary means by which humans communicate, but its evolution defies scientific explanation. A central aspect of (nonhuman) great ape vocal capacities that could advance the understanding of (spoken) language evolution relates to voice control. While traditionally presumed uniquely human, there is currently a growing volume of multidisciplinary data evidencing voice control in great apes^1–8^. To seek agreement between historical and modern views, here, through an innovative method – the use of a membranophone as a diagnostic tool for voice control in primates – we demonstrate that novel vocalizations in orangutans are indeed the product of vocal fold action and that orangutans can adjust their voice frequency and duration features rapidly and meaningfully. Therefore, voice control in great apes is only different from humans’ in degree, not kind.

The idea that great apes have “little to say” about language evolution dates back to great ape language projects that, nearly half a century ago, reared individuals in language-embedded settings and “home-labs” in an attempt to teach great apes to talk^9,10^. These great apes appeared to fail the task, whereas their achievements using gestures to communicate with humans were much more impressive^10,11^. The original researchers rearing and working with these apes explicitly stated, nevertheless, that their subjects did produce human words, such as, “cup”, “papa” and “mama”^9,12^ (https://www.youtube.com/watch?v=ab59zcsV35k). While subjects clearly underperformed compared with human children exposed to similar levels of spoken language, at face value, these historical studies suggest that the capacity for vocal production control in great apes may not be as limited as our own understanding of it (for a more comprehensive evaluation of these studies, see^1^). Solving the puzzle of language evolution, however, rests on this knowledge. Better understanding great ape vocal capacities will not contradict hypotheses that the human vocal tract evolved in unique ways to make speech production more efficient and effective^13^, but will instead reveal exactly what was the ancestral state and original hominid communicative capacities that were targeted by natural selection within the human clade and that ultimately laid path for the emergence and evolution of language.

Since the historical great ape language projects, various studies have started to reveal more nuanced and richer means through which primates can indeed control and modify their calls’ acoustics^14–18^, means that traditional ideas glossed over or failed to recognise altogether. Despite this crescendo of evidence for advanced vocal capacities, particularly in great apes^19–23^, including the production of novel vowel- and consonant-like calls^1,3,16–18,24–26^ (https://www.youtube.com/watch?v=ab59zcsV35k and https://www.youtube.com/watch?v=Lg50_1RSc0E&t=2s), orthodox beliefs that our closest relatives are poor models for language evolution persist^27,28^. These ideas endure on the claim of absence of evidence, specifically, evidence for the capacity to voluntary control the voice (i.e., vocal fold oscillation rate) – *active voicing* – which in human speech proto-typically underpins vowel-production.

[In the literature, production of new, modified or learned calls has been generally defined as vocal (production) learning. This term allows for cross-taxa comparisons^29^, regardless the specific mechanism(s) through which each species/taxon may actually achieve vocal learning. For instance, song birds do so through syrinx control, dolphins through blowhole control. Here, however, our primary goal was to establish a homologous link between mechanisms for voiced vocal production among hominids, namely between humans and great apes. Active voicing refers, hence, to a specific utilization of vocal control in the primate order through which individuals may achieve vocal learning. No attempt should thus be attempted (based on this study) to compare primate vocal capacities vs. taxa with different vocal anatomies].

Because active voicing is more difficult to empirically demonstrate in great apes than other primates on ethical grounds (e.g. as has been done through social isolation^30,31^ or parental interaction experiments^32,33^ with marmosets^14^), here, we provide a new line of evidence in great apes using an innovative approach – the use of a membranophone as a diagnostic tool for active voicing. We gave the opportunity to orangutans to use a kazoo – a membranophone widely available commercially – while a human demonstrated its use. This musical instrument was chosen because it is strictly activated by the player’s voice. Unlike a flute or a whistle, blowing does not produce sound, in which case the airflow closes up and immobilises the membrane of the instrument (hence, the instrument’s original slogan, “Remember, don’t blow – hum!”). Moreover, its correct operation requires the closing of the jaw and lip adjustment around the mouthpiece, an articulatory position that differs from other typical species-specific primate vocalisations, which typically involve open mouth (including typical vowel production and innate vocalizations in humans).

## Methods

To investigate active voicing in great apes, we worked with 6 orangutans (*Pongo*, 5 hybrids + 1 *P. abelli*) at Simon Skjodt International Orangutan Center in Indianapolis Zoo, Indiana, USA. Subjects were matched by sex-age class: two flanged males (Azy, Studbook ID:1616; Charly, 2655), two adult females (Knobi, 1733; Katy, 2248), and two unflanged males (Rocky, 3331; Basan, 3006). At the start of the trials, one subject in each class voluntarily produced a novel voiced call, or “vocalization”. Azy produced “home” vocalizations (Supplementary Video), Knobi “hug” vocalizations (Supplementary Video), and Rocky “wookies”^17^. The individuals were known to produce these unique vocalizations for several years prior as a means to gather the attention of and interact with human caretakers, but these vocalizations’ inception and development is unknown. These vocalizations were distinct from any call described in the orangutan repertoire^34^ and several individual-specific species-atypical vocalizations in orangutans (including wookies) have indeed been quantitatively confirmed to differ from anything produced in the wild^16,17^. For these three individuals, we verbally requested the production of their novel vocalizations into the kazoo (i.e. gazing directly the individuals, asking their participation, motivating and persuading the correct positioning of their lips on the mouthpiece and activation of the kazoo; e.g. see Supplementary Video) and by concurrently demonstrating the use of the kazoo. Context and settings remained constant at all times, thus, kazoo activation did not require change of communicative intent or function. No exercises of vocal mimicry or imitation were involved, and we only sought the activation of the kazoo by the subjects to confirm active voicing, whichever fashion it was possible for the individuals. Katy produced several novel voiceless calls (i.e., not engaging the voice), such as raspberries, smacks, splutters, clicks. She was, thus, included together with Charly and Basan in the group who did not produce novel vocalizations. For these three individuals, we also verbally requested them to vocalize into the kazoo but without the possibility of prompting, capturing or targeting a specific voluntary vocalization.

**Figure 1:**
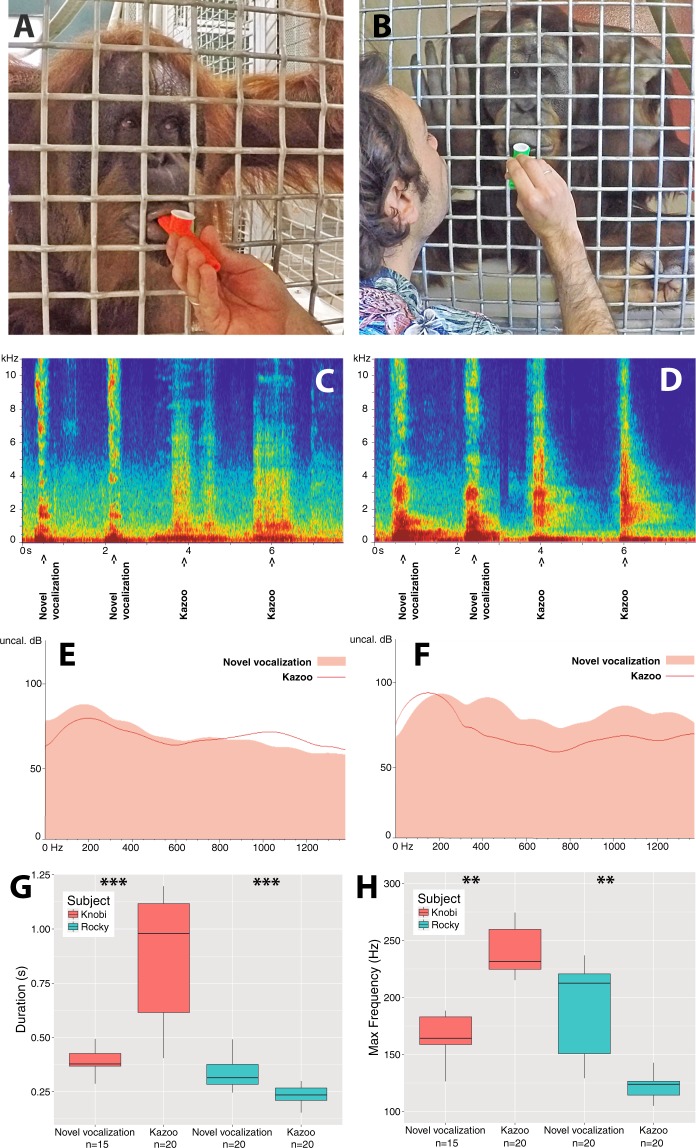
Knobi (**A**) and Rocky (**B**) during kazoo trials; Spectrogram (**C**,**D**), and power spectra (**E**,**F**) of respective novel vocalizations and kazoo pulses (Supplementary Audio); Acoustic differences in duration (**E**) and maximum frequency (**F**) between novel vocalization and kazoo pulses for each subject.

All subjects were individually presented with the same protocol and received similar exposure time, viz. ~55 min/subject across ~6 sessions/subject, with a minimum of 12 attempts provided per session. All individuals had never been trained in vocal exercises with or without instruments, with the exception of Rocky who has been previous involved in do-as-I-do vocal imitation games (for the duration of one hour over the last decade^17^) but who has never had vocal training with the use of sound instruments. All subjects were initially allowed to freely manipulate and investigate the kazoo. This, however, inexorably resulted in the destruction of the kazoo, at which point the experimenter held a new kazoo for the subject for the remaining session time and subsequent sessions (see Supplementary Video). The up-holding of the kazoo by the experimenter was made in a way to simply provide free access to the mouth piece of the kazoo. This was done without exerting any pressure or force in order to allow the individuals to be able to adjust the position and activation of the kazoo to their lips as needed. Because the activation of the kazoo’s membrane is independent from the angle of the mouthpiece at the lips, no effect by the experimenter was physically possible. Moreover, access to the kazoo’s mouthpiece was provided through a 1 × 1-inch mesh (Fig. 1), which meant the angle of the mouthpiece at the lips could not vary widely within and between trials and sessions. Food rewards (e.g. raisins, plumbs; always kept constant within trial sessions and part of subjects’ routine diet) were prepared by staff and provided non-differentially [i.e. regardless of (un)successful kazoo activation] during trials.

We collected audio and video recordings using a ZOOM H4next Handy recorder’s built-in microphones (ZOOM Corporation, Tokyo) and a GoPro Hero4 (San Mateo, California), respectively, at ~0.5 m away through a mesh separating humans and subjects at all times. Raven (version 1.5.0, Cornell Lab of Ornithology, Ithaca) was used for acoustic measures. Permutation tests were conducted in R^35^ and Bayesian Mann-Whitney U Tests were conducted with JASP (Version 0.9.1, University of Amsterdam). For acoustic comparisons, we used the first 15–20 vocalizations produced at the start of the study without kazoo and the final 20 vocalizations produced at the end of the study with kazoo.

## Result and Discussion

During trials, Rocky and Knobi activated the membranophone (Supplementary Audio and Video) by producing their novel vocalizations (already produced at the start of the study) into the kazoo, notably, within 11 and 34 minutes, respectively. Rocky activated the membranophone with ingressive airflow (as his novel vocalization^17^), while Knobi did so with egressive airflow (as her novel vocalization; Supplementary Video), indicating that kazoo activation was not a pure innovation or a result of parallel learning but an extension of their original vocalizations. Successful kazoo activation validated that both individuals deployed voluntary vocal fold oscillation for the production of novel vocalizations, thus, providing conclusive evidence for active voicing in orangutans.

Azy produced a novel vocalization but was unsuccessful in adjusting the jaw and lips to the instrument’s mouthpiece because his novel vocalization entailed tilting the head and a fast open-close mouth movement (Supplementary Video). During Katy’s trials, she attempted to produce voiceless calls into the instrument but to no avail, since active voicing was obligatory. Charly and Basan only succeeded in blowing air through the kazoo, which did not result in the instrument’s activation.

Beyond expectation, however, acoustic comparison of the novel vocalizations (produced before successful kazoo activation) by Rocky and Knobi vs. their respective kazoo pulses (produced at the end of the trials) revealed significant differences in duration (s) (Permutation test, r = 5000; Rocky: 0.25 percentile, p = 0; median, p = 0.0002, 0.75 percentile, p = 0.0004; Knobi: p = 0.0002, p = 0.0024, p = 0) and dominant frequency (Hz; that of highest dB) (Permutation test: Rocky, p = 0, p = 0, p = 0; Knobi, p = 0.0014, p = 0.005, p = 0) (Fig. 1, Supplementary Material). We recognize that our sample size was relatively small. Therefore, to verify the results, we replicated the comparison using Bayesian Mann-Whitney U Tests. Results confirmed a meaningful difference in the time and frequency parameters between the novel vocalizations and their respective kazoo pulses (Knobi; duration: BF_10_ = 492.27, W = 5; max frequency: BF_10_ = 46.45, W = 58 and Rocky; duration: BF_10_ = 94.16, W = 44; max frequency: BF_10_ = 507.34, W = 35.5). As indicated by BF values, the hypothesis that acoustic parameters underwent a non-negligible change as a result of experience in the use of the kazoo was 492 and 94 times (for duration) and 46 and 507 times (for max frequency) more likely to explain the results than the null hypothesis (i.e. no difference in parameters).

**Figure 2:**
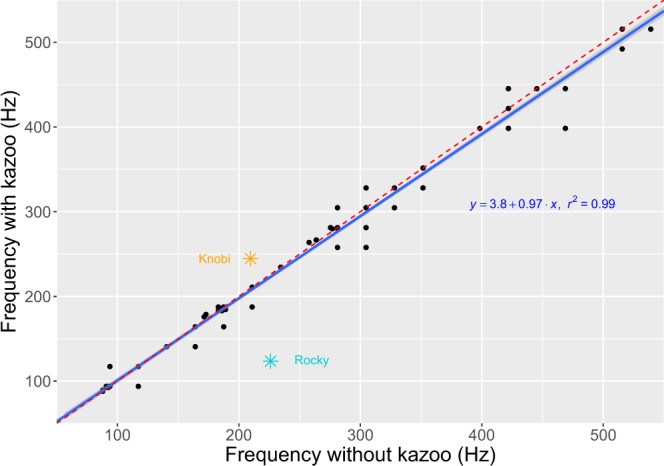
Kazoo frequency response test (blue) showing no shift of voice input. Dashed line (red) is has intercept = 0 and slope = 1 and is provided for comparison purposes only. Star symbols (orange and cyan) depict the average frequency difference of each individual’s novel vocalizations vs. corresponding kazoo activation, which lay well beyond the narrow 95% confidence interval margin (grey) of the kazoo’s frequency response line.

To establish any potential influence of the vibratory properties of the kazoo’s membrane on the observed differences, we tested the kazoo’s frequency responses (Supplementary Audio). Tests revealed a flat response line. That is, the kazoo directly mirrored the voice frequency’s input, with kazoo’s output frequency imposing virtually no shift (Fig. 2). These results were not unexpected since the use of a membranophone as a general musical instrumental would otherwise be impracticable and equivalent to playing an instrument constantly out of tune. A kazoo’s membrane vibrates at equal frequency as the vocal folds and is, thus, inherently tuned to the player’s voice. Figure 2 shows that the voice differences observed in Rocky and Knobi lay far beyond what the kazoo’s vibratory properties could have generated by itself.

These remarkably quick (<1 hour) and significant changes in the two subjects prove that experience with a kazoo induced plastic adjustment of airflow (as indicated by duration differences) and vocal fold oscillation rate (as indicated by frequency differences). These modifications probably allowed individuals to activate the kazoo more easily. Critically, each individual did so in opposite fashion from the other: one *increased* her voice duration and frequency, whereas the other *decreased* his voice duration and frequency. This observation confirms a role of individual learning. Rocky has previously shown to control his voice intonation to match a human’s modulating voice^17^. Our results confirm that he indeed achieved this through active voicing. Simultaneously, by describing this capacity in a non-related adult female, our results show that this capacity is not exclusive to an odd case and is most likely latent-yet-mobilizable in the genus.

As in the classic example made famous by Nassim Taleb^36^, the observation of one black swan is sufficient to prove that not all swans are white. Likewise, our results are adequate and sufficient to prove that active voicing is not absent in great apes, even though for now this has only been demonstrated in two (unrelated) subjects. It is worth noticing that we invested less than an hour of research effort per subject. Future studies investing more time may, thus, seek to reveal the level of prevalence and the correlates of active voicing among great apes. Our results give a preliminary but strong indication that each individual’s background and level of vocal proficiency at the time of testing are paramount factors to take in consideration in the implementation and interpretation of results. This suggests that interventions and training with great apes should probably follow similar guidelines and considerations as when working with children with specific language impairments who require protocols specific to their  individual difficulties. None of the orangutans who did not produced novel vocalizations was able to activate the kazoo, indicating that more effort time and different (i.e. easier) types of exercises will likely be required to raise these individuals’ skill of vocal control to the level required to activate a kazoo. It should be noticed, however, that producing novel vocalizations was not sufficient for the use of the kazoo as a diagnostic tool. For example, the specific mechanics and movement of Azy’s novel vocalization did not enable the use of the kazoo. In this case, more effort time may be required to teach this individual to use the kazoo adequately, whereas vocal-training per se may not be required. A renewed research interest and effort in surveying and facilitating vocal production learning in captive great apes (including via active voicing) in a wider scale will gradually improve our understanding of how and why great apes engage in novel vocal production, as well as how to best tailor different training and experimental protocols to different individuals. This will help match test difficulty with subjects’ acquired vocal proficiency level and, ultimately, avoid false negatives – as was likely the case with historical great ape language projects^1^.

Our results are consistent with the description of call cultures and vocal innovation in wild orangutans^23,34,37,38^. Together, captive and wild evidence suggests that individual’s acoustic environment may help to shape their call repertoire. For example, it is known that retired orangutans from the entertainment industry tend to bring with them unique individual-specific calls never heard in the wild or elsewhere in captivity^16–18^. However, it has also been described various times that captive orangutans in accredited zoos can learn to imitate human sounds from their unaware caretakers, with no training or explicit ape-directed behaviour^18^. Thus, captive life does not *always* translate in a poorly stimulating acoustic environment for the individuals, but alas, this is probably the case for the majority of individuals. The knowledge that certain orangutans – if not all – may have a latent capacity for vocal production learning warrants the implementation of new sound stimuli and new types of human-ape interaction protocols in captivity to enrich the communicative and cognitive lives of these individuals. In the case of captive orangutans who have been rescued and confiscated from the pet trade but who can be reintroduced back into the wild, these individuals may require vocal and communicative training as part of their rehabilitation before release. Indeed, recent findings suggest that vital aspects of survival in the forest may be transmitted vocally from mother to infant^22^. Thus, a similar teaching role could be carried out by the caring staff at orangutan rehabilitation centres and forest-schools.

## Conclusions

Our results provide the first positive diagnostic test of vocal production learning in great apes, namely active voicing, during novel voiced vocal production in orangutans. Membranophone activation was successful within minutes after first exposure, precluding that it developed out of training or conditioning. Indeed, within one hour of practice, individuals tailored their voice duration and frequency insomuch that kazoo pulses showed major differences to the novel vocalizations from which they originally derived (Figs 1 and 2). Our results are, thus, qualitatively different from laboratory work with monkeys that, after months-long intensive conditional training and reflex-building, produced sub-variants of the same innate vocalization type^39^.

The observation that orangutans can exercise active voicing and have a degree of online control over the larynx suggests that the roots of this ability within the human clade are much older than previously thought. This finding helps resolve a long-standing polarizing debate in the field. It gives categorical support that fundamental voice control to produce new vowel-like and other new voiced calls is shared among humans and great apes^1^, even if the exact voice parameters/quality may have differed from extinct hominids depending on soft tissue anatomic differences in the vocal apparatus. Alongside a fast and ongoing multidisciplinary shift in our understanding of great ape vocal faculties^1,5–7^, this study demonstrates that behavioural *prima materia* for the evolution of articulate language was present among ancient members of the Homonidea family.

### Ethics

This study was approved by the reseach and ethics board of the Indianapolis Zoo. All experiments were performed in accordance with relevant guidelines and regulations.

## Supplementary information

Supplementary Audio File 1

Supplementary Audio File 2

Supplementary Audio File 3

Supplementary Video File 1

Supplementary Video File 2
